# A scaling limit theorem for controlled branching processes with a size-divisible term

**DOI:** 10.1007/s13398-026-01883-9

**Published:** 2026-06-16

**Authors:** Miguel González, Pedro Martín-Chávez, Inés del Puerto

**Affiliations:** 1 Department of Statistics, University of Warwick, CV4 7AL Coventry, UK; 2 Departamento de Matemáticas, Universidad de Extremadura, 06006 Badajoz, Spain; 3 Instituto de Computación Científica Avanzada, Universidad de Extremadura, 06006 Badajoz, Spain

**Keywords:** Branching process, Weak convergence, Infinitesimal generator, Martingale problem, Limit theorem, Scaling limit, 60J80, 60F05

## Abstract

This paper establishes general sufficient conditions for a sequence of controlled branching processes to converge weakly on the Skorokhod space. We focus on a class of control mechanisms that extend previous results by decomposing those random variables into the sum of two independent components: an immigration term, which depends on the current population size, and a size-divisible term, which can be expressed as the sum of random contributions from each individual. This extension allows us to capture a broad range of control functions including Poisson, binomial, and negative binomial distributions, commonly used in the literature. The assumptions are formulated in terms of probability generating functions of the offspring and control laws, distinguishing in this latter between the immigration and the size-divisible parts. The limit process is shown to be a continuous-state branching process with dependent immigration. The proof essentially relies on tightness arguments and the identification of a martingale problem. We also identify the special case in which the limit reduces to a classical Feller branching diffusion with immigration.

## Introduction

Controlled branching processes (CBPs) are a class of discrete-time, discrete-state branching processes that arise as a modification of the classical Galton–Watson processes (GWPs). Widely used in population dynamics, they are a very general family of models characterized by the fact that the number of parents in each generation is determined by random variables known as control functions $\{\phi ^{(n)}(j)\}$. This random mechanism is interpreted as the impact of the environment on reproduction law and is the key feature of the process that gives it its great versatility. The model was introduced by [18] but with deterministic control functions, the extension to random control functions is due to [22]. A comprehensive discussion of most of the results available in the literature for CBPs can be found in the following monograph [8].

The question to be addressed in this article is the study of the convergence of rescaled sequences of CBPs in the Skorokhod space. This problem is sometimes referred to in the literature as scaling limit theorems or high-density limit theorems. It has been considered by many authors for several branching models. For instance, it is well known that rescaled GWPs converge to continuous-time, continuous-state branching processes. This statement can be found in the works of [11] and [9]. In fact, they generalise a previous result of [5], who obtained the so-called Feller branching diffusion as the limit process. Something similar happens when immigration comes into play. Scaling limits of Galton–Watson processes with immigration result in continuous-state branching processes with immigration, as shown by [10] or [13]. See also [21] for a Feller branching diffusion with immigration. However, the problem is far from being completely solved in the case of CBPs. The first contributions in this sense were diffusion approximation results in the works by [6, 7, 19], but only a partial answer to the question is given.

Recently, [17] showed a scaling limit of CBPs whose control functions can be written as $\phi ^{(n)}(j) = j + \psi ^{(n)}(j)$, where $\psi ^{(n)}(j)$ are non-negative integer valued random variables. It turns out that the limiting process is a continuous-state process with dependent immigration (CSBPDI). It is the purpose of this paper to extend those results by Liu to control variables such that the difference $\phi ^{(n)}(j)-\psi ^{(n)}(j)$ may be also a non-negative random variable $\varphi ^{(n)}(j)$, not necessarily the deterministic case $\varphi ^{(n)}(j) = j.$ In line with Liu’s work, we call $\psi ^{(n)}(j)$ the immigration term. In turn, we will denote $\varphi ^{(n)}(j)$ as the size-divisible term because we will assume that these random variables are *j*-divisible. In essence, this means that they can be expressed as a sum of *j* random variables. A more precise definition will be given later. The extension explained in this paragraph will allow dealing with more general control variables than those proposed by Liu as explained in Sect. 4.

Apart from the aforementioned hypothesis, convergence conditions for the generating probability functions (PGFs) of the reproduction law and the immigration term are required. In addition to divisibility, smoothness conditions are assumed for the size-divisible term.

Besides this introduction, the structure of the paper is as follows. Section 2 mathematically defines the stochastic models of the present work, that is, CBPs and CSBPDIs. In Sect. 3 we rigorously outline the problem to be solved as well as the hypotheses assumed in the article and the statement of the main theorem. Section 4 is dedicated to a discussion comparing our contribution with previous works on this problem. We also include some examples of control functions to which the results can be applied. In Sect. 5 we collect all the results of the paper that lead to the proof of the main theorem. Finally, an Appendix contains the proof of an auxiliary lemma.

## Probability models

### Controlled branching process

Let $(\Omega , \mathcal {F}, \mathbb {P})$ be the probability space where all the random variables of this paper are defined. Positive integers and non-negative integers are denoted by $\mathbb {N}$ and $\mathbb {N}_0$, respectively. Consider a family of independent and identically distributed (IID) random variables $\{X^{n,i}: n\in \mathbb {N}_0,i\in \mathbb {N}\}$ taking values in $\mathbb {N}_0$ and with PGF *g*. Let $\{\phi ^{(n)}(j): n\in \mathbb {N}_0\}$ be an IID environment also taking values in $\mathbb {N}_0$ and with PGF $c^{(j)}$ for each $j\in \mathbb {N}_0$. The distributions of $\{X^{n,i}: n\in \mathbb {N}_0,i\in \mathbb {N}\}$ and $\{\phi ^{(n)}(j): n\in \mathbb {N}_0\}$ are called, respectively, the offspring and the control distributions. It is assumed independence between the offspring and control distributions. Given a $\mathbb {N}_0$-valued random variable *Z*(0) independent of $\{X^{n,i},\phi ^{(n)}(j):n,j\in \mathbb {N}_0,i\in \mathbb {N}\}$, a CBP is defined inductively as

$$\begin{aligned} Z(n+1) = \sum _{i=1}^{\phi ^{(n)}(Z(n))}X^{n,i}, \qquad n\in \mathbb {N}_0, \end{aligned}$$

with the convention $\sum _{i=1}^0 = 0.$ Then the discrete (time and state) Markov chain $\{Z(n): n\in \mathbb {N}_0\}$ is completely characterized by the random initial generation *Z*(0), the offspring PGF *g* and the control PGFs $\{c^{(j)}: j\in \mathbb {N}_0\}$, using the standard terminology.

The intuitive interpretation says that *Z*(*n*) denotes the size of the *n*-th generation of the population and $X^{n,i}$ is the offspring size of the *i*-th progenitor in the *n*-th generation, where the total number of progenitors is given by $\phi ^{(n)}(Z(n))$.

Lastly, we will denote by $(Q(i,j): i, j\in \mathbb {N}_0)$ the one-step transition matrix of $\{Z(n): n\in \mathbb {N}_0\}$, whose definition follows from

1 $$\begin{aligned} \sum _{j=0}^\infty Q(i,j)s^j = \mathbb {E}\left[ g(s)^{\phi ^{(n)}(i)}\right] = c^{(i)}(g(s)), \qquad s\in [0,1]. \end{aligned}$$

We refer the reader to [8] for further details.

### Continuous-state branching process with dependent immigration

According to [14], a CSBPDI is a Markov process in $[0,\infty )$ with generator $\mathcal {L}$ defined by

$$\begin{aligned} \mathcal {L}f(x) =&- a x f'(x) + \frac{1}{2} b^2 x f''(x) + x \int _0^\infty [f(x+y) - f(x) - y f'(x)] \mu (\textrm{d}y) \\&+ \alpha (x) f'(x) + \int _0^\infty [f(x+y)-f(x)]r(x,y) \nu (\textrm{d}y), \qquad f \in C_c^2([0,\infty )), \end{aligned}$$

where $C_c^2([0,\infty ))$ is the set of bounded continuous real functions on $[0,\infty )$ with compact support and with bounded continuous derivatives up to the second order, $a\in \mathbb {R}$ and $b\in [0,\infty )$ are constants, $\mu$ and $\nu$ are $\sigma$-finite measures on $(0,\infty )$ with

$$\begin{aligned} \int _0^\infty \left( u\wedge u^2\right) \mu (\textrm{d}u)<\infty \quad \text{ and } \quad \int _0^\infty \left( 1\wedge u\right) \nu (\textrm{d}u) <\infty , \end{aligned}$$

and $\alpha$ and *r* are non-negative Borel functions on $[0,\infty )$ and $[0,\infty )\times (0,\infty )$ such that satisfy, respectively:

*Linear growth condition.* There exists a constant $K\ge 0$ verifying

2 $$\begin{aligned} \alpha (x) + \int _0^\infty r(x,y) y \nu (\textrm{d}y ) \le K(1+x), \qquad x\in [0,\infty ). \end{aligned}$$

*Yamada–Watanabe type condition.* There exists a non-decreasing concave function $\beta :[0,\infty )\rightarrow [0,\infty )$ verifying $\int _{0+} \beta (y)^{-1} \textrm{d}y = \infty$ and

$$\begin{aligned} |\alpha (x) -\alpha (y)| + \int _0^\infty | r(x,z)-r(y,z)|z\nu (\textrm{d}z) \le \beta (|x-y|), \qquad x,y\in [0,\infty ). \end{aligned}$$

Alternatively, there is an equivalent way of defining such processes using stochastic integral equations, see [14, Theorem 5.4]. Let $(\Omega , \mathcal {F}, \mathcal {F}_t, \mathbb {P})$ be a filtered probability space under the usual hypothesis. Let us consider a standard $(\mathcal {F}_t)$-Wiener process $(\mathcal {W}(t):t\in [0,\infty ))$, a $(\mathcal {F}_t)$-Poisson random measure $M(\textrm{d}s, \textrm{d}u, \textrm{d}z)$ on $(0,\infty )^3$ with intensity $\textrm{d}s\mu (\textrm{d}u)\textrm{d}z$ and a $(\mathcal {F}_t)$-Poisson random measure $N(\textrm{d}s, \textrm{d}u,\textrm{d}z)$ on $(0,\infty )^3$ with intensity $\textrm{d}s\nu (\textrm{d}u)\textrm{d}z$. Denoting by $\tilde{M}(\textrm{d}s, \textrm{d}u, \textrm{d}z) = M(\textrm{d}s, \textrm{d}u, \textrm{d}z) - \textrm{d}s\mu (\textrm{d}u)\textrm{d}z$ the corresponding compensated measure of *M*, the CSBPDI can be characterized as the non-negative pathwise solution of

$$\begin{aligned} y(t)&= y(0) + \int _0^t [\alpha (y(s)) - ay(s)]\textrm{d}s + b\int _0^t \sqrt{y(s)}\, \textrm{d}\mathcal {W}(s) \nonumber \\&\quad + \int _0^t\int _0^\infty \int _0^{y(s-)} u\tilde{M}(\textrm{d}s,\textrm{d}u,\textrm{d}z) + \int _0^t\int _0^\infty \int _0^{r(y(s-),u)} uN(\textrm{d}s,\textrm{d}u,\textrm{d}z). \end{aligned}$$

With the previous notation, for each triple $(a,b,\mu )$, we define a function *G* called branching mechanism given by

3 $$\begin{aligned} G(\lambda ) = a\lambda + \frac{1}{2}b^2\lambda ^2 + \int _0^\infty \left( \textrm{e}^{-\lambda u}-1+\lambda u\right) \mu (\textrm{d}u), \qquad \lambda \in [0,\infty ). \end{aligned}$$

Likewise, for each triple $(\alpha ,\nu , r)$, we introduce another function *H* defined as

4 $$\begin{aligned} H(x,\lambda ) = \alpha (x) \lambda + \int _0^\infty \left( 1-\textrm{e}^{-\lambda u}\right) r(x,u)\nu (\textrm{d}u), \qquad x,\lambda \in [0,\infty ). \end{aligned}$$

We called this second function as dependent immigration mechanism.

## Setup and main results

Once the models discussed in the paper have been introduced, we properly formulate the problem to be addressed and our main contribution.

Let us consider a sequence of CBPs $\{Z_k(n): n\in \mathbb {N}_0\}_{k\in \mathbb {N}}$ built from a sequence of offspring functions $\{g_k\}_{k\in \mathbb {N}}$ and a sequence of control functions $\{c_k^{(j)}: j\in \mathbb {N}_0\}_{k\in \mathbb {N}}$. For each $k\in \mathbb {N}$, $\{k^{-1} Z_k(n): n\in \mathbb {N}_0\}$ is a discrete Markov chain with state space $k^{-1} \mathbb {N}_0 = \{k^{-1}n: n\in \mathbb {N}_0\}$. Let $\{\gamma _k\}_{k\in \mathbb {N}}$ be a sequence of positive real numbers, then for each $k\in \mathbb {N}$ we introduce a continuous-time stochastic process $(z_k(t): t\in [0,\infty ))$ given by

5 $$\begin{aligned} z_k(t) = \frac{1}{k} Z_k(\lfloor \gamma _k t \rfloor ), \qquad t\in [0,\infty ). \end{aligned}$$

Therefore, for all $k\in \mathbb {N}$ we defined a process whose paths belong to the space of non-negative càdlàg functions over the half-line, $D([0,\infty ))$, endowed with the Skorohod topology, and where the half-line $[0,\infty )$ is endowed with the Euclidean distance. The goal in this work is to establish the weak convergence on $D([0,\infty ))$ of such processes $z_k$ as $k\rightarrow \infty$. Under certain assumptions, the limit process will be a CSBPDI.

For each $k\in \mathbb {N}$ and $n,j\in \mathbb {N}_0$, the starting point is that every control variable $\phi _k^{(n)}(j)$ may be decomposed as $\phi _k^{(n)}(j) = \varphi _k^{(n)}(j) + \psi _k^{(n)}(j)$, i.e. as the sum of two $\mathbb {N}_0$-valued random variables. Furthermore, properties of independence and same distribution are inherited. That is, we suppose that these new random variables are independent of each other, $\{\varphi _k^{(n)}(j):n\in \mathbb {N}\}$ are identically distributed for each $k\in \mathbb {N}$, $j\in \mathbb {N}_0$ and $\{\psi _k^{(n)}(j):n\in \mathbb {N}\}$ are also identically distributed for each $k\in \mathbb {N}$, $j\in \mathbb {N}_0$. The reader can summarize this paragraph as Assumption 0.

### Assumption 1

There exists a constant $\gamma _0\in [0,\infty )$ such that $\lim _{k\rightarrow \infty } \gamma _k = \infty$ and $\lim _{k\rightarrow \infty } \gamma _k / k = \gamma _0.$

Above condition imposes that the time scaling sequence is always divergent but with a growth rate *O*(*k*), using Landau notation.

### Assumption 2

There exists a constant $m\in (0,\infty )$ such that the sequence of functions $\{G_k\}_{k\in \mathbb {N}}$ is uniformly Lipschitz on each bounded interval and converges uniformly on each bounded interval as $k\rightarrow \infty$, where

6 $$\begin{aligned} G_k(\lambda ) = \frac{k\gamma _k}{m}\left[ g_k\bigg (1-\frac{\lambda }{k}\bigg )-\left( 1-\frac{m\lambda }{k}\right) \right] , \qquad \lambda \in [0,k]. \end{aligned}$$

### Remark 1

From Assumption 2 it follows that there exists a locally Lipschitz function $G:[0,\infty )\rightarrow \mathbb {R}$ such that $G_k\rightarrow G$ as $k\rightarrow \infty$ uniformly on each bounded interval. In particular, *G* is continuous.

### Remark 2

Under Assumption 2, the sequence of functions

$$\begin{aligned} \frac{\textrm{d}G_k}{\textrm{d}\lambda }(\lambda ) = \gamma _k \left[ 1-\frac{1}{m} \frac{\textrm{d}g_k}{\textrm{d}s}\bigg (1-\frac{\lambda }{k}\bigg )\right] ,\qquad \lambda \in [0,k], \end{aligned}$$

is uniformly bounded on each bounded interval, so there exists a positive constant $K_2>0$ such that

7 $$\begin{aligned} \gamma _k \left| 1-\frac{1}{m} \frac{\textrm{d}g_k}{\textrm{d}s}(1-)\right| \le K_2, \qquad k\in \mathbb {N}. \end{aligned}$$

Therefore, for eack $k\in \mathbb {N}$, the mean of the reproduction law (or offspring mean) is finite and, by Assumption 1, the derivative of the offspring PGFs verifies

$$\begin{aligned} \frac{\textrm{d}g_k}{\textrm{d}s} \bigg (1-\frac{\lambda }{k}\bigg )\rightarrow m, \qquad \text {as } k\rightarrow \infty , \end{aligned}$$

uniformly on each bounded interval. As a consequence, the candidate for being *m* is precisely the limit of the convergent sequence of offspring means.

The only restriction imposed on the offspring distribution is Assumption 2. Similar conditions have been previously assumed by other authors, cf. [13] and [17], but they always force $m=1$, which for us is not necessary. This allows us to work with reproduction laws whose offspring mean sequence converges to a number other than 1.

Next, for each $k\in \mathbb {N}$ and $j\in \mathbb {N}_0$, let us denote by $h_k^{(j)}$ the PGF of the identically distributed random variables $\{\psi _k^{(n)}(j):n\in \mathbb {N}\}$.

### Assumption 3

For each $c_1,c_2\in [0,\infty )$, the sequence of functions $\{H_k\}_{k\in \mathbb {N}}$ converges to the dependent immigration mechanism *H* defined in (4) uniformly on $[0,c_1]\times [0,c_2]$ as $k\rightarrow \infty$, where

8 $$\begin{aligned} H_k(x,\lambda ) = \gamma _k\left[ 1-h_k^{(\lfloor kx \rfloor )}\bigg (1-\frac{\lambda }{k}\bigg )\right] , \qquad x\in [0,\infty ),\quad \lambda \in [0,k]. \end{aligned}$$

### Assumption 4

There exists a constant $K_1>0$ such that

$$ \left| \frac{\partial H_k}{\partial \lambda }( x,0)\right| =\frac{\gamma _k}{k} \frac{\textrm{d} h_k^{(\lfloor k x\rfloor )}}{\textrm{d} s} (1-) \le K_1(1+x), \qquad x \in [0,\infty ), \quad k \in \mathbb {N}. $$

Respect to the conditions over $\varphi _k^{(n)}(j)$, we introduce formally the concept of *j*-divisibility ($j\in \mathbb {N}_0$) for random variables.

### Definition 1

A random variable *Y* is called *j*-divisible ($j\in \mathbb {N}_0$) if it may be expressed as the sum of *j* IID random variables. By convention we establish that 0-divisible means *Y* is identically null.

The reader should note that a particular case is any infinitely divisible distribution, i.e. one that is *j*-divisible for all $j\in \mathbb {N}$. To delve deeper into the divisibility of random variables, see e.g. [20].

The next condition can be seen as the most relevant in our paper and will play a key role in the proofs of the main results.

### Assumption 5

For all $k\in \mathbb {N}$ and $n,j\in \mathbb {N}_0$, the random variable $\varphi _k^{(n)}(j)$ is *j*-divisible.

CBPs with *j*-divisible control functions and their link with population-size-dependent branching processes have been recently studied in [3].

### Remark 3

For all $k\in \mathbb {N}$, the control PGFs may be written as $c_k^{(0)} = h_k^{(0)}$ and

$$\begin{aligned} c_k^{(j)} = \left[ f_k^{(j)}\right] ^j h_k^{(j)}, \qquad j\in \mathbb {N}, \end{aligned}$$

where $f_k^{(j)}$ is the *j*-th root of the PGF of $\varphi _k^{(n)}(j)$.

Apart from the divisibility property on $\varphi _k^{(n)}(j)$, we suppose also the following condition on the functions $f_k^{(j)}$.

### Assumption 6

There exist constants ${\varrho }\in \mathbb {R}$ and ${\varsigma }\in [0,\infty )$ such that

9 $$\begin{aligned} \lim _{k\rightarrow \infty } \sup _{j\in \mathbb {N}, \lambda \in [0,M]} \left| F_{k}^{(j)} (\lambda ) - F(\lambda )\right| = 0, \qquad \text {for each } M>0, \end{aligned}$$

where $F(\lambda ) = \varrho \lambda + \frac{1}{2}\varsigma ^2\lambda ^2$ for all $\lambda \in [0,\infty )$ and

$$\begin{aligned} F_{k}^{(j)} (\lambda ) = k \gamma _k \left[ f_k^{(j)}\bigg (1-\frac{\lambda }{k}\bigg )-\left( 1-\frac{\lambda }{mk}\right) \right] , \qquad \lambda \in [0,k], \qquad k,j\in \mathbb {N.} \end{aligned}$$

Further, we assume that the functions $\{F_{k}^{(j)}\}_{k,j\in \mathbb {N}}$ are uniformly Lipschitz on each bounded interval.

### Remark 4

Under the Lipschitz condition in Assumption 6, the functions

$$\begin{aligned} \frac{\textrm{d}F_{k}^{(j)}}{\textrm{d}\lambda }(\lambda ) = \gamma _k \left[ \frac{1}{m}-\frac{\textrm{d}f_k^{(j)}}{\textrm{d}s}\bigg (1-\frac{\lambda }{k}\bigg )\right] ,\qquad \lambda \in [0,k], \end{aligned}$$

are uniformly bounded on each bounded interval, so there is a positive and finite constant given by

10 $$\begin{aligned} K_3 = \sup _{k,j\in \mathbb {N}} \left| \gamma _k \left( 1- m \frac{\textrm{d}f_k^{(j)}}{\textrm{d}s}(1-)\right) \right| . \end{aligned}$$

Finally, we need an hypothesis on the initial generations $z_k(0)$. To be precise, the uniform boundedness of the first moment is required.

### Assumption 7

$$\sup _{k\in \mathbb {N}} \mathbb {E} [z_k(0)]<\infty .$$

Having introduced all the working hypotheses, we can now present the main result of the paper.

### Theorem 1

Let $(z_k(t): t\in [0,\infty ))$ be the sequence defined in (5) and suppose that Assumptions 1–7 hold. If $z_k(0)$ converges in distribution to *z*(0) as $k\rightarrow \infty$, then $(z_k(t): t\in [0,\infty ))$ converges in distribution on $D([0,\infty ))$ to $(z(t):t\in [0,\infty ))$, which is the pathwise unique solution of

11 $$\begin{aligned} z(t)&= z(0) + \int _0^t [m\alpha (z(s))-(a+{\varrho m}) z(s)]\textrm{d}s + \int _0^t \sqrt{(b^2+{ \varsigma ^2 m^2} )z(s)}\, \textrm{d}\mathcal {W}(s) \nonumber \\&\quad + \int _0^t\int _0^\infty \int _0^{z(s-)} u\tilde{M}(\textrm{d}s,\textrm{d}u,\textrm{d}z) + \int _0^t\int _0^\infty \int _0^{r(z(s-),m^{-1}u)} uN(\textrm{d}s,m^{-1}\textrm{d}u,\textrm{d}z), \end{aligned}$$

with initial value *z*(0). Therefore, $(z(t):t\in [0,\infty ))$ is a CSBPDI with branching mechanism

$$\begin{aligned} \lambda \in [0,\infty )\longmapsto G(\lambda ) + { F(m\lambda )}, \end{aligned}$$

dependent immigration mechanism

$$\begin{aligned} (x,\lambda )\in [0,\infty )^2\longmapsto H(x, m\lambda ), \end{aligned}$$

and generator given by

12 $$\begin{aligned} Af(x)&= [m \alpha (x) - (a+{\varrho m}) x]f'(x) + \frac{1}{2}(b^2+{ \varsigma ^2 m^2}) xf''(x) \nonumber \\&\quad + x \int _0^\infty \left[ f(x+y)-f(x)-yf'(x)\right] \mu (\textrm{d}y) \nonumber \\&\quad + \int _0^\infty \left[ f(x+y)-f(x)\right] r(x,m^{-1}y) \nu ( m^{-1} \textrm{d}y), \qquad f\in C_c^2([0,\infty )). \end{aligned}$$

As a particular case of the previous theorem, we state the following corollary, which is interesting due to its relationship with other results in the literature, as will be explained in the next section.

### Corollary 2

Under the same hypotheses as Theorem 1 and maintaining the notation, if $G(\lambda )=a\lambda +\frac{1}{2}b^2\lambda ^2$ and $H(x,\lambda )=\alpha \lambda$ with $\alpha$ being a constant, then the limit process $(z(t): t\in [0,\infty ))$ is a Feller branching diffusion with immigration and can be characterized as the

solution of the stochastic equation

$$\begin{aligned} z(t) = z(0) + \alpha m t - (a+{\varrho m})\int _0^t z(s)\textrm{d}s + \int _0^t \sqrt{(b^2+{ \varsigma ^2 m^2})z(s)}\, \textrm{d}\mathcal {W}(s), \end{aligned}$$

where $(\mathcal {W}(t):t\in [0,\infty ))$ is a standard Wiener process. Furthermore, its generator is given by

$$\begin{aligned} A f(x) = [\alpha m - (a+{\varrho m}) x]f'(x) + \frac{1}{2}(b^2+{ \varsigma ^2 m^2}) xf''(x) , \qquad f\in C_c^2([0,\infty )). \end{aligned}$$

## Discussion on known results and examples

In this section, we compare our results with previous ones on literature for CBPs. According to our knowledge, to date the only papers addressing the problem of scaling limit theorems for CBPs are [6, 7, 17, 19].

The first three references prove the weak convergence in the Skorokhod space of a rescaled sequence of CBPs towards a diffusion process. Said diffusion process is nothing more than a Feller branching diffusion with immigration whose generator in the three cases can be written as

$$\begin{aligned} Lf(x)=(C_1+C_2 x)f'(x)+C_3 x f''(x), \qquad f\in C_0^2([0,\infty )), \end{aligned}$$

where $C_1,C_2,C_3$ are real constants and $C_1,C_3$ are non-negative. Therefore, the limit process satisfies the stochastic differential equation

$$\begin{aligned} \textrm{d}W(t) = (C_1+C_2 W(t))\textrm{d}t + \sqrt{2C_3 W(t)}\, \textrm{d}\mathcal {W}(t), \end{aligned}$$

where $(\mathcal {W}(t):t\in [0,\infty ))$ is a standard Wiener process. The set of hypotheses necessary to establish the above convergence differs markedly from the assumptions here, those hypotheses cannot be rewritten exactly in terms of these ones nor vice versa. In those references, conditions are assumed on the moments of both the reproduction law and the control distribution, while here we work more in terms of PGFs. Corollary 2 can be seen as a counterpart result to [19, Lemma 1], [6, Theorem 1] and [7, Theorem 3.1].

Regarding the recent publication [17], it is a work closely related to this paper addressing the same problem, the study of the convergence of the sequence of scaled CBPs $(z_k(t): t\in [0,\infty ) )$, see (5). Here we generalize it. Let us remember at this point Assumption 0, which allows us to write the control variables as the sum respectively of a size-divisible term and an immigration term, $\phi _k^{(n)}(j) = \varphi _k^{(n)}(j) + \psi _k^{(n)}(j)$. Liu’s starting hypothesis is similar assuming $\phi _k^{(n)}(j) = j + \psi _k^{(n)}(j)$, that is, he imposes $\varphi _k^{(n) }(j) = j.$ However, the big disadvantage of its starting hypothesis is that the result is not applicable to most of the control functions used in the literature, see e.g. [8, pp. 68, 129]. We solved this issue by allowing $\varphi _k^{(n) }(j)$ to be those random variables satisfying Assumptions 5 and 6. As we will see below, these conditions are not that restrictive and allows treating typical control variables such as Poisson, binomial or negative binomial distributions. This is the strength of Theorem 1 compared to [17, Theorem 4.2].

Finally, we include in this section a series of possible valid random variables for the size-divisible term to apply the main theorem.

- *Poisson control*. It is well known that the Poisson distribution is infinitely divisible, see e.g. [20, p. 28], so it satisfies Assumption 5. For convenience we write $\varphi _k^{(n)}(j) \sim \text {Poisson} (r_k(j)j)$, $j\in \mathbb {N}$, so that it is easy to see that Assumption 6 is satisfied if there exists ${\varrho }$ such that
  13 $$\begin{aligned} \lim _{k\rightarrow \infty } \sup _{j} \left| \gamma _k\left[ 1 - m r_k(j)\right] - {\varrho m} \right| = 0. \end{aligned}$$
  Indeed, taking $\varsigma ^2 = \gamma _0 m^{-2}$, it is easy to see that Assumption 6 follows from the following inequality,
  $$\begin{aligned} \left| F_{k}^{(j)} (\lambda ) - F(\lambda )\right|&= \left| k \gamma _k \left[ \textrm{e}^{-r_k(j) \lambda / k} - \left( 1-\frac{\lambda }{mk}\right) \right] - \left( \varrho \lambda + \frac{1}{2}\gamma _0m^{-2}\lambda ^2\right) \right| \\&\le \frac{\left| \gamma _k \left[ 1 - m r_k(j) \right] - \varrho m \right| }{m} \lambda + \left| \frac{\gamma _k r_k(j)^2 }{2k} - \frac{\gamma _0}{2m^2} \right| \lambda ^2 + \frac{\gamma _k r_k(j)^3}{6 k^2} \lambda ^3 . \end{aligned}$$
  For example, let
  $$r_k(j) = \frac{1}{m}\left( 1-\frac{2}{k}+\frac{1}{jk\log k}\right) , \qquad k\ge 2, \quad j\in \mathbb {N},$$
  and ${\varrho } = 2\gamma _0 {m^{-1}}$, the reader can check that (13) holds.
- *Binomial control*. The binomial distribution trivially satisfies Assumption 5 as it is the sum of Bernoulli distributions. Now we conveniently write $\varphi _k^{(n)}(j) \sim \text {Binomial} (N_k(j)j,p_k(j))$ with $N_k(j)\in \mathbb {N}$ and $p_k(j)\in [0,1]$ for $j\in \mathbb {N}$. Suppose that there exist ${\varrho }\in \mathbb {R}$ and $p_0\in [0,1]$ such that
  $$\begin{aligned} \lim _{k\rightarrow \infty } \sup _{j} \left| \gamma _k\left[ 1 - m N_k(j)p_k(j)\right] - {\varrho m} \right| = 0, \end{aligned}$$
  $$\begin{aligned} \lim _{k\rightarrow \infty } \sup _{j} \left| p_k(j) - p_0 \right| = 0, \end{aligned}$$
  then Assumption 6 holds with $\varsigma ^2=\gamma _0 m^{-1}(m^{-1}-p_0)$. Indeed, using the PGF of the binomial distribution, Assumption 6 follows easily taking into account that
  $$\begin{aligned}&\left| F_{k}^{(j)} (\lambda ) - F(\lambda )\right| = \left| k \gamma _k \left[ \left( 1 - \frac{p_k(j) \lambda }{k} \right) ^{N_k(j)} - \left( 1- \frac{\lambda }{mk}\right) \right] - \left( \varrho \lambda + \frac{1}{2}\gamma _0m^{-1}(m^{-1}-p_0)\lambda ^2\right) \right| \\&\quad \le \frac{\left| \gamma _k \left[ 1 - m N_k(j) p_k(j) \right] - \varrho m \right| }{m} \lambda + \left| \frac{\gamma _k N_k(j) (N_k(j) -1) p_k(j)^2 }{2k} - \frac{\gamma _0\frac{1}{m}(\frac{1}{m}-p_0)}{2} \right| \lambda ^2 \\&\qquad + \frac{\gamma _k N_k(j)^3 p_k(j)^3}{6 k^2} \lambda ^3 . \end{aligned}$$
  For instance, let
  $$\begin{aligned} N_k(j)=1+jk(k+1), \qquad p_k(j) = \frac{1}{mjk^2}, \qquad k,j\in \mathbb {N}, \end{aligned}$$
  ${\varrho }=-\gamma _0 { m^{-1}}$ and $p_0 =0$ as possible choices.
- *Negative Binomial control*. As in the first case, the negative binomial distribution is infinitely divisible (the reader can consult the same reference as before). If $\varphi _k^{(n)}(j) \sim \text {NegativeBinomial} (N_k(j)j,p_k(j))$ with $p_k(j)\in (0,1]$ for $j\in \mathbb {N}$ and
  $$\begin{aligned} \lim _{k\rightarrow \infty } \sup _{j} \left| \gamma _k\left[ 1 - m N_k(j)\frac{1-p_k(j)}{p_k(j)}\right] - {\varrho m} \right| = 0, \end{aligned}$$
  $$\begin{aligned} \lim _{k\rightarrow \infty } \sup _{j} \left| \frac{1-p_k(j)}{p_k(j)} - q_0 \right| = 0, \end{aligned}$$
  where ${\varrho }\in \mathbb {R}$ and $q_0\in [0,\infty )$, then Assumptions 5 and 6 hold with $\varsigma ^2 = \gamma _0 m^{-1}(m^{-1}+q_0)$. Indeed, proceeding as before, the claim follows from the following inequality
  $$\begin{aligned} \left| F_{k}^{(j)} (\lambda ) - F(\lambda )\right|&= \left| k \gamma _k \left[ \left( 1 + \frac{1-p_k(j)}{p_k(j)}\frac{\lambda }{k} \right) ^{-N_k(j)} - \left( 1- \frac{\lambda }{mk}\right) \right] \right. \\&\quad \left. - \left( \varrho \lambda + \frac{1}{2}\gamma _0m^{-1}(m^{-1}+q_0)\lambda ^2\right) \right| \\&\le \frac{\left| \gamma _k \left[ 1 - m N_k(j) \frac{1-p_k(j)}{p_k(j)} \right] - \varrho m \right| }{m} \lambda \\&\quad + \left| \frac{\gamma _k N_k(j) (N_k(j) +1) \frac{(1-p_k(j))^2}{p_k(j)^2} }{2k} - \frac{\gamma _0\frac{1}{m}(\frac{1}{m}+q_0)}{2} \right| \lambda ^2 \\&\quad + \frac{\gamma _k N_k(j) (N_k(j)+1)(N_k(j)+2) \left( \frac{1-p_k(j)}{p_k(j)}\right) ^3}{6 k^2} \lambda ^3 . \end{aligned}$$
  Let us remember that a particular case of the negative binomial distribution is the geometric distribution. A possible example for the latter is given by choosing
  $$\begin{aligned} N_k(j)=1, \qquad p_k(j) = \frac{m\exp [(j+k)^{-2}]}{1+m\exp [(j+k)^{-2}]}, \qquad k,j\in \mathbb {N}, \end{aligned}$$
  $${\varrho }=0$$ and $$q_0 =m^{-1}$$.

## Proof of the Theorem 1

The proof is inspired on the ideas given in [17]. The fact of introducing the size-divisible term in the control variables requires a non-trivial extra work. We emphasize here this part in some auxiliary results needed to the proof of Theorem 1.

We begin this section with a lemma that sheds light on the limit function of Remark 1, which arose from the convergence of the sequence in Assumption 2. Lemma 3 is close to [12, Corollary 1] without being exactly the same result. For the ease of the reader, we include the proof details in Appendix 1.

### Lemma 3

If Assumption 2 holds, then the limit function *G* in Remark 1 has representation (3), i.e. *G* is a branching mechanism.

Given a branching mechanism, it is always possible to build a sequence such that Assumption 2 holds, as the next result claims. We can think on this lemma as the converse, in some sense, of the above one. The proof is also postponed for Appendix 1.

### Lemma 4

For any function *G* with representation (3) there is a sequence of functions $\{G_k\}_{k\in \mathbb {N}}$ satisfying Assumption 2.

Next, we provide three propositions associated with the offspring and control PGFs. They will be useful later in proving the convergence of the generators of scaled sequence defined in (5).

Let us introduce then two sequences of functions, $\{S_k\}_{k\in \mathbb {N}}$ and $\{T_k\}_{k\in \mathbb {N}}$, related to the offspring PGFs. For $k\in \mathbb {N},$ wet set

14 $$\begin{aligned} S_k(\lambda ) = \frac{k\gamma _k}{m} \left[ g_k(\textrm{e}^{-\lambda /k}) - \left( 1-\frac{m\lambda }{k}\right) \right] , \qquad \lambda \in [0,\infty ), \end{aligned}$$

15 $$\begin{aligned} T_k(\lambda ) = k\left[ 1-g_k(\textrm{e}^{-\lambda /k})\right] , \qquad \lambda \in [0,\infty ). \end{aligned}$$

### Proposition 5

Under Assumptions 1 and 2,

16 $$\begin{aligned} \lim _{k\rightarrow \infty } S_k(\lambda ) = G(\lambda ) + \frac{1}{2}\gamma _0\lambda ^2, \end{aligned}$$

17 $$\begin{aligned} \lim _{k\rightarrow \infty } T_k(\lambda ) = m\lambda , \end{aligned}$$

both uniformly on each bounded interval.

### Proof

Let us begin with (16) fixing $l\in (0,\infty )$. There exists $k_0 \in \mathbb {N}$ such that $k_0\ge l$. For all $\lambda \in [0,l]$ and $k\in \mathbb {N}$ with $k\ge k_0$, the following equality holds

$$\begin{aligned} S_k(\lambda ) = G_k(\lambda ) + \frac{k\gamma _k}{m} \left[ g_k(\textrm{e}^{-\lambda /k}) - g_k\bigg (1-\frac{\lambda }{k}\bigg )\right] , \end{aligned}$$

and, by Assumption 2, it is enough to show that

18 $$\begin{aligned} \lim _{k\rightarrow \infty } \sup _{\lambda \in [0,l]} \left| \frac{k\gamma _k}{m} \left[ g_k(\textrm{e}^{-\lambda /k}) - g_k\bigg (1-\frac{\lambda }{k}\bigg )\right] - \frac{1}{2}\gamma _0\lambda ^2\right| = 0. \end{aligned}$$

Applying the mean value theorem, there exists $\xi _k(\lambda )\in [1-\lambda /k,\textrm{e}^{-\lambda /k}]\subset [1-l/k,1]$ such that

$$\begin{aligned} \frac{k\gamma _k}{m} \left[ g_k(\textrm{e}^{-\lambda /k}) - g_k\bigg (1-\frac{\lambda }{k}\bigg )\right] = \frac{k\gamma _k}{m} \left[ \textrm{e}^{-\lambda /k} - \left( 1-\frac{\lambda }{k}\right) \right] \frac{\textrm{d}g_k}{\textrm{d}s}(\xi _k(\lambda )), \end{aligned}$$

and, from the Taylor theorem with the Lagrange remainder,

$$\begin{aligned} \textrm{e}^{-\lambda /k} - \left( 1-\frac{\lambda }{k}\right) = \frac{1}{2 k^2}\lambda ^2 - \frac{\textrm{e}^{-\eta _k /k}}{6k^3}\lambda ^3, \end{aligned}$$

for $\eta _k \in [0,l]$. Then the following inequalities hold for all $\lambda \in [0,l]$,

$$\begin{aligned}&\left| \frac{k\gamma _k}{m} \left[ g_k(\textrm{e}^{-\lambda /k}) - g_k\bigg (1-\frac{\lambda }{k}\bigg )\right] - \frac{\gamma _0\lambda ^2}{2}\right| \\&\qquad \le \frac{1}{2}\left| \frac{\gamma _k}{mk} \frac{\textrm{d}g_k}{\textrm{d}s}(\xi _k(\lambda )) - \gamma _0 \right| \lambda ^2 + \frac{\gamma _k\textrm{e}^{-\eta _k /k} \lambda ^3}{6mk^2} \frac{\textrm{d}g_k}{\textrm{d}s}(\xi _k(\lambda )) \\&\qquad \le \frac{1}{2}\left( \frac{\gamma _k}{k} \left| \frac{1}{m}\frac{\textrm{d}g_k}{\textrm{d}s}(\xi _k(\lambda )) - 1\right| + \left| \frac{\gamma _k}{k}- \gamma _0 \right| \right) l^2 + \frac{\gamma _k l^3}{6mk^2} \frac{\textrm{d}g_k}{\textrm{d}s}(1-) . \end{aligned}$$

Notice the use of $\textrm{d}g_k /\textrm{d}s$ as a non-negative monotonically increasing function. Thus, taking into account Assumption 1 and Remark 2,

$$\begin{aligned} \lim _{k\rightarrow \infty } \sup _{\lambda \in [0,l]} \frac{1}{2}\left( \frac{\gamma _k}{k} \left| \frac{1}{m}\frac{\textrm{d}g_k}{\textrm{d}s}(\xi _k(\lambda )) - 1\right| + \left| \frac{\gamma _k}{k}- \gamma _0 \right| \right) l^2 = 0, \end{aligned}$$

$$\begin{aligned} \lim _{k\rightarrow \infty } \frac{\gamma _k l^3}{6mk^2} \frac{\textrm{d}g_k}{\textrm{d}s}(1) = 0, \end{aligned}$$

and, as a result, (18) is proven.

Finally, (17) follows trivially from the next equality

$$\begin{aligned} T_k(\lambda ) = m\lambda - \frac{m}{\gamma _k}S_k(\lambda ), \end{aligned}$$

for all $$\lambda \in [0,\infty ).$$ $$\square $$

Next proposition includes what the interaction is like between the offspring PGF and the PGF of the immigration term $\psi _k^{(n)}(j)$. This is where Assumption 3 comes into play.

### Proposition 6

Under Assumptions 1–3,

19 $$\begin{aligned} \lim _{k\rightarrow \infty } \gamma _k\left[ 1-h_k^{(\lfloor kx \rfloor )}\big (g_k(\textrm{e}^{-\lambda /k})\big )\right] = H(x,m\lambda ), \end{aligned}$$

uniformly on $[0,c_1]\times [0,c_2]$ for any $c_1,c_2\in [0,\infty )$.

### Proof

Recalling (15), the function $T_k$ takes values in [0, *k*], so the composition $H_k (x, T_k(\lambda ))$ is well defined for all $x,\lambda \in [0,\infty )$. In fact, for all $k\in \mathbb {N}$,

$$\begin{aligned} H_k(x, T_k(\lambda )) = \gamma _k\left[ 1-h_k^{(\lfloor kx \rfloor )}\big (g_k(\textrm{e}^{-\lambda /k})\big )\right] , \qquad x, \lambda \in [0,\infty ). \end{aligned}$$

By Assumption 3, the sequence $H_k$ converges uniformly to *H* as $k\rightarrow \infty$ on $[0,c_1]\times [0,c_2]$. Hereby, this proposition follows trivially from (17). $\square$

The last proposition corresponds to an auxiliary limit that involve the functions $f_k^{(j)}$ (see Assumption 5 and Remark 3 to recall the connection with the size-divisible term of the control variables).

### Proposition 7

Under Assumptions 1, 2, 5 and 6,

20 $$\begin{aligned} \lim _{k\rightarrow \infty } \sup _{j\in \mathbb {N}} \left| \gamma _k\left[ 1-\frac{\log f_k^{(j)}\big (g_k(\textrm{e}^{-\lambda /k})\big ) }{f_k^{(j)}\big (g_k(\textrm{e}^{-\lambda /k})\big )-1}\right] + \frac{1}{2} \gamma _0\lambda \right| = 0, \end{aligned}$$

uniformly on each bounded interval.

### Proof

First, we show that

21 $$\begin{aligned} \lim _{k\rightarrow \infty } \sup _{j\in \mathbb {N}} \left| \gamma _k\left[ f_k^{(j)}\big (g_k(\textrm{e}^{-\lambda /k})\big ) - 1\right] + \gamma _0\lambda \right| = 0. \end{aligned}$$

Recalling the definitions of $S_k$ and $T_k$ from equations (14) and (15), we can write

22 $$\begin{aligned} f_k^{(j)}\big (g_k(\textrm{e}^{-\lambda /k})\big ) -1&= \frac{1}{k\gamma _k} F_{k}^{(j)}(T_k(\lambda )) - \frac{T_k(\lambda )}{mk} \nonumber \\&= \frac{1}{k\gamma _k} \left[ F_{k}^{(j)}(T_k(\lambda )) + S_k(\lambda ) \right] - \frac{\lambda }{k}. \end{aligned}$$

From Proposition 5 and Assumption 6, we see that $F_{k}^{(j)}(T_k(\lambda )) + S_k(\lambda )$ converges to $F(m\lambda )+G(\lambda )+\frac{1}{2}\gamma _0\lambda ^2$ as $k\rightarrow \infty$, so that

$$ \gamma _k\left[ f_k^{(j)}\big (g_k(\textrm{e}^{-\lambda /k})\big ) - 1\right] + \gamma _0\lambda = \frac{F_{k}^{(j)}(T_k(\lambda )) + S_k(\lambda ) }{k} + \left( \gamma _0-\frac{\gamma _k}{k}\right) \lambda , $$

vanishes in the limit verifying (21).

Recall now that there exists $\eta \in [x,1]$ such that $\log x = x-1 - \frac{(x-1)^2}{2}+\frac{(x-1)^3}{3\eta ^3}$ for all $x\in (0,1].$ Therefore,

$$\begin{aligned} 1-\frac{\log f_k^{(j)}\big (g_k(\textrm{e}^{-\lambda /k})\big )}{f_k^{(j)}\big (g_k(\textrm{e}^{-\lambda /k})\big )-1} = \frac{f_k^{(j)}\big (g_k(\textrm{e}^{-\lambda /k})\big )-1}{2} - \frac{\left[ f_k^{(j)}\big (g_k(\textrm{e}^{-\lambda /k})\big )-1\right] ^2}{3[\zeta _k^{(j)}(\lambda )]^3}, \end{aligned}$$

where $\zeta _k^{(j)}(\lambda )\in [f_k^{(j)}\big (g_k(\textrm{e}^{-\lambda /k})\big ),1]$. From the previous expression, we get

$$\begin{aligned} \left| \gamma _k\left[ 1-\frac{\log f_k^{(j)}\big (g_k(\textrm{e}^{-\lambda /k})\big ) }{f_k^{(j)}\big (g_k(\textrm{e}^{-\lambda /k})\big )-1}\right] + \frac{1}{2} \gamma _0\lambda \right|&\le \left| \frac{\gamma _k\left[ f_k^{(j)}\big (g_k(\textrm{e}^{-\lambda /k})\big )-1\right] }{2} + \frac{\gamma _0\lambda }{2} \right| \\&\quad + \frac{\gamma _k\left[ f_k^{(j)}\big (g_k(\textrm{e}^{-\lambda /k})\big )-1\right] ^2}{3[f_k^{(j)}\big (g_k(\textrm{e}^{-\lambda /k})\big )]^3}. \end{aligned}$$

Namely, (20) is deduced because both terms on the right hand side converge to 0 due to (21). $\square$

Let us recall at this point the generator given in (12). For $f\in C_c^2([0,\infty ))$, we have

$$\begin{aligned} Af(x)&= [m \alpha (x) - (a+{\varrho m}) x]f'(x) + \frac{1}{2}(b^2+{ \varsigma ^2 m^2}) xf''(x) \nonumber \\&\quad + x \int _0^\infty \left[ f(x+y)-f(x)-yf'(x)\right] \mu (\textrm{d}y) \nonumber \\&\quad + \int _0^\infty \left[ f(x+y)-f(x)\right] r(x,m^{-1}y) \nu ( m^{-1} \textrm{d}y), \end{aligned}$$

and let us denote by $A_k f =\gamma _k(\tilde{A}_k f -f)$ the (discrete) generator of the scaled process $(z_k(t): t \in [0, \infty ))$, where

$$\begin{aligned} \tilde{A}_k f(x) =\mathbb {E}\left[ f(k^{-1}Z_k(n+1))\mid k^{-1}Z_k(n)=x\right] . \end{aligned}$$

For each $\lambda \in [0,\infty )$, we write

$$\begin{aligned} e_\lambda :x\in [0,\infty )\longmapsto \textrm{e}^{-\lambda x}. \end{aligned}$$

### Remark 5

It is proved in [17, Lemma 2.2 ] that the linear span of $\{e_{\lambda }: \lambda \ge 0\}$ is dense in $C_c^2([0,\infty ))$ in the sense of pointwise convergence.

Next theorem establishes the approximation between the generator of the scaled process, $A_k$, and the one of the CSBPDI, *A*. In view of Remark 5, we just prove the convergence for the negative exponential functions.

### Theorem 8

Under Assumptions 1–6,

23 $$\begin{aligned} \lim _{k\rightarrow \infty } \sup _{x\in k^{-1}\mathbb {N}_0} \left| A_k e_{\lambda }(x)-A e_{\lambda }(x) \right| = 0, \end{aligned}$$

for all $$\lambda \in [0,\infty ).$$

### Proof

Firstly, let us apply the operator in (12) to the negative exponential functions. We get

$$\begin{aligned} A e_\lambda (x)&= -[m \alpha (x) - (a+{\varrho m}) x]\lambda \textrm{e}^{-\lambda x} + \frac{1}{2}(b^2+{ \varsigma ^2 m^2}) x \lambda ^2 \textrm{e}^{-\lambda x} \\&\quad + x \textrm{e}^{-\lambda x} \int _0^\infty (\textrm{e}^{-\lambda y} - 1+ \lambda y) \mu (\textrm{d}y) \\&\quad + \textrm{e}^{-\lambda x} \int _0^\infty (\textrm{e}^{-\lambda y} - 1) r(x,m^{-1}y) \nu ( m^{-1} \textrm{d}y) \\&= \textrm{e}^{-\lambda x} \left[ x \left( G(\lambda ) + {F(m\lambda )} \right) - H(x,m\lambda ) \right] . \end{aligned}$$

Recalling (1) and taking into account that for all $k\in \mathbb {N}$, $\{k^{-1} Z_k(n): n\in \mathbb {N}_0\}$ is a discrete Markov chain with state space $k^{-1} \mathbb {N}_0$, it is easy to see that the corresponding one-step transition probabilities are characterized by

$$\begin{aligned} \tilde{A}_k e_\lambda (x) =\mathbb {E} \left[ \textrm{e}^{-\lambda k^{-1} Z_k(n+1)} \bigm | k^{-1} Z_k(n)=x\right] = c_k^{(kx)}\big (g_k(\textrm{e}^{-\lambda /k})\big ). \end{aligned}$$

Hence, the generator $A_k$ is

$$\begin{aligned} A_k e_\lambda (x) = \gamma _k \left[ c_k^{(kx)}\big (g_k(\textrm{e}^{-\lambda /k})\big ) - \textrm{e}^{-\lambda x} \right] . \end{aligned}$$

For $\lambda =0,$ (23) trivially holds because $Ae_\lambda$ and $A_ke_\lambda$ are identically null.

Let us fix $\lambda >0$ and $\lambda _0\in (0,\lambda )$. In particular, $A_k e_\lambda (0) = \gamma _k \left[ h_k^{(0)}\big (g_k(\textrm{e}^{-\lambda /k})\big ) - 1 \right]$ which converges to $Ae_\lambda (0)=- H(0,m\lambda )$ as $k\rightarrow \infty$ by Proposition 6. Then, in order to prove (23), it is enough to compute supreme for $x\in k^{-1}\mathbb {N}$ because we have already seen that the case $x=0$ holds. In what follows, we restrict ourselves to the case $x\in k^{-1}\mathbb {N}$.

Now Assumption 5 plays a key role. We can write

$$\begin{aligned} A_k e_\lambda (x) = \gamma _k \left( \left[ f_k^{(kx)}\big (g_k(\textrm{e}^{-\lambda /k})\big )\right] ^{kx} h_k^{(kx)}\big (g_k(\textrm{e}^{-\lambda /k})\big ) - \textrm{e}^{-\lambda x} \right) = B_k(x,\lambda ) + C_k(x,\lambda ), \end{aligned}$$

where

24 $$\begin{aligned} B_k(x,\lambda )&= \gamma _k \left[ f_k^{(kx)}\big (g_k(\textrm{e}^{-\lambda /k})\big )\right] ^{kx} \left( h_k^{(kx)}\big (g_k(\textrm{e}^{-\lambda /k})\big ) - 1 \right) , \nonumber \\ C_k(x,\lambda )&= \gamma _k \left( \left[ f_k^{(kx)}\big (g_k(\textrm{e}^{-\lambda /k})\big )\right] ^{kx} - \textrm{e}^{-\lambda x} \right) . \end{aligned}$$

We will prove first that

25 $$\begin{aligned} \lim _{k\rightarrow \infty } \sup _{x\in k^{-1}\mathbb {N}} \textrm{e}^{\lambda _0 x} \left| C_k(x,\lambda ) - x \textrm{e}^{-\lambda x} \left( G(\lambda ) + {F(m\lambda )} \right) \right| = 0. \end{aligned}$$

Indeed,

$$\begin{aligned} C_k(x,\lambda )&= \textrm{e}^{-\lambda x} \gamma _k \left( \left[ \textrm{e}^{\lambda /k} f_k^{(kx)}\big (g_k(\textrm{e}^{-\lambda /k})\big )\right] ^{kx} - 1 \right) \\&= \textrm{e}^{-\lambda x} \gamma _k \left[ \exp \bigg ( kx \log \left[ \textrm{e}^{\lambda /k} f_k^{(kx)}\big (g_k(\textrm{e}^{-\lambda /k})\big )\right] \bigg ) - 1 \right] , \end{aligned}$$

and denoting

26 $$\begin{aligned} U_k(x,\lambda ) = k\gamma _k \log \left[ \textrm{e}^{\lambda /k} f_k^{(kx)}\big (g_k(\textrm{e}^{-\lambda /k})\big )\right] , \end{aligned}$$

we apply Taylor’s theorem,

$$\begin{aligned} C_k(x,\lambda )&= \textrm{e}^{-\lambda x} \gamma _k \left( \exp \left[ \frac{xU_k(x,\lambda ) }{\gamma _k} \right] - 1 \right) \\&= \textrm{e}^{-\lambda x} \left( xU_k(x,\lambda ) \right) + \textrm{e}^{-\lambda x}\gamma _k \frac{1}{2}\textrm{e}^{\zeta _k(x,\lambda )} \left[ \frac{xU_k(x,\lambda )}{\gamma _k} \right] ^2, \end{aligned}$$

where $\zeta _k(x,\lambda )$ is real number between 0 and $\gamma _k^{-1}[xU_k(x,\lambda ) ]$. Therefore, in order to prove (25), it is sufficient to show that

27 $$\begin{aligned} \lim _{k\rightarrow \infty } \sup _{x\in k^{-1}\mathbb {N}} \left| U_k(x,\lambda ) - \left( G(\lambda ) + {F(m\lambda )} \right) \right| = 0, \end{aligned}$$

28 $$\begin{aligned} \lim _{k\rightarrow \infty } \sup _{x\in k^{-1}\mathbb {N}} \frac{1}{2}\textrm{e}^{\lambda _0x+\zeta _k(x,\lambda )-\lambda x} \frac{\left[ xU_k(x,\lambda )\right] ^2}{\gamma _k} = 0. \end{aligned}$$

Let us focus first in (27). Introducing

29 $$\begin{aligned} W_k(x,\lambda ) = \frac{\log \left[ f_k^{(kx)}\big (g_k(\textrm{e}^{-\lambda /k})\big ) \right] }{f_k^{(kx)}\big (g_k(\textrm{e}^{-\lambda /k})\big ) - 1}, \end{aligned}$$

and recalling (22), we obtain

30 $$\begin{aligned} U_k(x,\lambda )&= k\gamma _k \left( \frac{\lambda }{k} + \log \left[ f_k^{(kx)}\big (g_k(\textrm{e}^{-\lambda /k})\big )\right] \right) \nonumber \\&= k\gamma _k \left( \frac{\lambda }{k} + W_k(x,\lambda )\left[ f_k^{(kx)}\big (g_k(\textrm{e}^{-\lambda /k})\big ) - 1\right] \right) \nonumber \\&= W_k(x,\lambda )\left[ F_{k}^{(kx)}(T_k(\lambda )) + S_k(\lambda ) \right] + \gamma _k \left( 1 - W_k(x,\lambda )\right) \lambda . \end{aligned}$$

Thus (27) is equivalent to prove the following limits

31 $$\begin{aligned} \lim _{k\rightarrow \infty } \sup _{x\in k^{-1}\mathbb {N}} \left| \gamma _k\left( 1-W_k(x,\lambda )\right) \lambda + \frac{1}{2} \gamma _0\lambda ^2 \right| = 0, \end{aligned}$$

32 $$\begin{aligned} \lim _{k\rightarrow \infty } \sup _{x\in k^{-1}\mathbb {N}} \left| W_k(x,\lambda )\left[ F_{k}^{(kx)}(T_k(\lambda )) + S_k(\lambda ) \right] - \left( F(m\lambda ) + G(\lambda ) + \frac{1}{2}\gamma _0\lambda ^2 \right) \right| = 0. \end{aligned}$$

Equation (31) follows directly from (20). In fact, (20) also implies

33 $$\begin{aligned} \lim _{k\rightarrow \infty } \sup _{x\in k^{-1}\mathbb {N}} |W_k(x,\lambda )-1|=0, \qquad \text {for all }\lambda >0. \end{aligned}$$

Equation (32) follows straightforwardly from Proposition 5, Assumption 6 and (33).

Finally, we show (28) to conclude the proof of (25). Since (27) holds, it is enough to see that

34 $$\begin{aligned} \lim _{k\rightarrow \infty } \sup _{x\in k^{-1}\mathbb {N}} x^2\textrm{e}^{\lambda _0x+\zeta _k(x,\lambda )-\lambda x} < \infty . \end{aligned}$$

Indeed, $$\zeta _k(x,\lambda ) < \max \left\{ 0, \gamma _k^{-1}[xU_k(x,\lambda )] \right\} $$, so

$$\begin{aligned} \zeta _k(x,\lambda )+ \lambda _0 x -\lambda x < \max \left\{ 0, \gamma _k^{-1}xU_k(x,\lambda )\right\} +\lambda _0 x-\lambda x. \end{aligned}$$

Recall that $U_k(x,\lambda )= \gamma _k \left( \lambda + k \log \left[ f_k^{(kx)}\big (g_k(\textrm{e}^{-\lambda /k})\big )\right] \right)$ converges according to (27). Then

$$\begin{aligned} \lim _{k\rightarrow \infty }\sup _{x\in k^{-1}\mathbb {N}}\left| k \log \left[ f_k^{(kx)}\big (g_k(\textrm{e}^{-\lambda /k})\big )\right] +\lambda \right| =0. \end{aligned}$$

Since, for *k* large enough

$$\begin{aligned} \zeta _k(x,\lambda )+\lambda _0 x -\lambda x< x \max \left\{ \lambda _0-\lambda , \lambda _0+k\log \left[ f_k^{(kx)}\big (g_k(\textrm{e}^{-\lambda /k})\big )\right] \right\} <0, \qquad \end{aligned}$$

we deduce (34) and the proof of (25) ends.

Now we prove that

35 $$\begin{aligned} \lim _{k\rightarrow \infty } \sup _{x\in k^{-1}\mathbb {N}}\left| \textrm{e}^{-\lambda x} H(x,m\lambda ) +B_k(x,\lambda )\right| = 0. \end{aligned}$$

Recalling the definition of $B_k(x,\lambda )$, we see that

$$\begin{aligned}&\left| \textrm{e}^{-\lambda x} H(x,m\lambda ) +B_k(x,\lambda )\right| \\&\quad = \left| \textrm{e}^{-\lambda x} H(x,m\lambda ) - \left[ f_k^{(kx)}\big (g_k(\textrm{e}^{-\lambda /k})\big )\right] ^{kx} H_k(x, T_k(\lambda )) \right| \\&\quad \le H(x,m\lambda )\left| \textrm{e}^{-\lambda x} - \left[ f_k^{(kx)}\big (g_k(\textrm{e}^{-\lambda /k})\big )\right] ^{kx}\right| \\&\quad \quad + \left[ f_k^{(kx)}\big (g_k(\textrm{e}^{-\lambda /k})\big )\right] ^{kx}\left| H(x,m\lambda ) - H_k(x, T_k(\lambda )) \right| \end{aligned}$$

Let us work on the terms of the previous expression. From condition (2) and equation (4), we see that

36 $$\begin{aligned} 0 \le H(x,\lambda ) \le K \lambda (1 + x), \qquad x, \lambda \in [0,\infty ). \end{aligned}$$

Introducing two new quantities

$$\begin{aligned} \epsilon _k(x,\lambda )&= \textrm{e}^{\lambda _0 x} [C_k(x,\lambda ) - x\textrm{e}^{-\lambda x} R(\lambda )], \\ R(\lambda )&= G(\lambda ) + {F(m\lambda )}, \end{aligned}$$

we deduce that

$$\begin{aligned} \left| \textrm{e}^{-\lambda x} - \left[ f_k^{(kx)}\big (g_k(\textrm{e}^{-\lambda /k})\big )\right] ^{kx}\right| = \gamma _k^{-1} |C_k(x,\lambda )| \le \frac{\textrm{e}^{-\lambda _0 x }|\epsilon _k(x,\lambda )| + x\textrm{e}^{-\lambda x} |R(\lambda )|}{\gamma _k}. \end{aligned}$$

Likewise, from (24),

$$\begin{aligned} 0 \le \left[ f_k^{(kx)}\big (g_k(\textrm{e}^{-\lambda /k})\big )\right] ^{kx} = \gamma _k^{-1}C_k(x,\lambda )+\textrm{e}^{-\lambda x} = \frac{\textrm{e}^{-\lambda _0 x }\epsilon _k(x,\lambda ) + x\textrm{e}^{-\lambda x} R(\lambda )}{\gamma _k} +\textrm{e}^{-\lambda x}. \end{aligned}$$

Let us fix $M>0$. We study (35) by first focusing on $x\in [0,M] \cap k^{-1} \mathbb {N}$ and next in $x\in (M,\infty ) \cap k^{-1} \mathbb {N}$. We have

$$\begin{aligned}&\sup _{x\in [0,M] \cap k^{-1}\mathbb {N}}\left| \textrm{e}^{-\lambda x} H(x,m\lambda ) +B_k(x,\lambda )\right| \\&\quad \le \sup _{x\in [0,M] \cap k^{-1}\mathbb {N}} \left[ K m \lambda (1 + x)\right] \frac{\textrm{e}^{-\lambda _0 x }|\epsilon _k(x,\lambda )| + x\textrm{e}^{-\lambda x} |R(\lambda )|}{\gamma _k} \\&\qquad + \sup _{x\in [0,M] \cap k^{-1}\mathbb {N}} \left( \frac{\textrm{e}^{-\lambda _0 x }\epsilon _k(x,\lambda ) + x\textrm{e}^{-\lambda x} R(\lambda )}{\gamma _k} +\textrm{e}^{-\lambda x}\right) \left| H(x,m\lambda ) - H_k(x, T_k(\lambda )) \right| . \end{aligned}$$

By Assumption 1, Proposition 6 and equation (25), we get

$$\begin{aligned} \lim _{k\rightarrow \infty } \sup _{x\in [0,M]\cap k^{-1}\mathbb {N}}\left| \textrm{e}^{-\lambda x} H(x,m\lambda ) +B_k(x,\lambda )\right| = 0. \end{aligned}$$

However, Proposition 6 is not helpful to handle $\left| H(x,m\lambda ) - H_k(x, T_k(\lambda )) \right|$ when $x\in (M,\infty ) \cap k^{-1}\mathbb {N}$. Therefore, an alternative strategy is needed. By mean value theorem and Assumption 4,

$$ 0 \le H_k(x,\lambda ) \le \lambda K_1(1+x), $$

so that, using the triangle inequality and (36), we have

$$\begin{aligned} \sup _{k \in \mathbb {N}} |H(x,m\lambda ) - H_k(x,T_k(\lambda ))|&\le K m\lambda (1 + x) + K_1(1+x) \sup _{k \in \mathbb {N}} T_k(\lambda ) \\&\le \textrm{e}^{\lambda _0 x} [K m\lambda + K_1\sup _{k \in \mathbb {N}} T_k(\lambda ) ]\sup _{x\ge 0} [(1+x)\textrm{e}^{-\lambda _0 x}], \end{aligned}$$

where clearly $\sup _{x\ge 0} [(1+x)\textrm{e}^{-\lambda _0 x}] <\infty$ and $\sup _{k \in \mathbb {N}} T_k(\lambda ) <\infty$ by (17). Thus,

$$\begin{aligned}&\sup _{x\in (M,\infty ) \cap k^{-1}\mathbb {N}}\left| \textrm{e}^{-\lambda x} H(x,m\lambda ) +B_k(x,\lambda )\right| \\&\quad \le \sup _{x\in (M,\infty ) \cap k^{-1}\mathbb {N}} \left[ K m \lambda (1 + x)\right] \frac{\textrm{e}^{-\lambda _0 x }|\epsilon _k(x,\lambda )| + x\textrm{e}^{-\lambda x} |R(\lambda )|}{\gamma _k} \\&\qquad + \sup _{x\in (M,\infty ) \cap k^{-1}\mathbb {N}} \left( \frac{\textrm{e}^{-\lambda _0 x }\epsilon _k(x,\lambda ) + x\textrm{e}^{-\lambda x} R(\lambda )}{\gamma _k} +\textrm{e}^{-\lambda x}\right) \textrm{e}^{\lambda _0 x}\\&\qquad \quad \;\; [K m\lambda + K_1\sup _{k \in \mathbb {N}} T_k(\lambda ) ]\sup _{x\ge 0} [(1+x)\textrm{e}^{-\lambda _0 x}]. \end{aligned}$$

Finally, from Assumption 1 and (25), we get

$$\begin{aligned} \varlimsup _{k\rightarrow \infty } \sup _{x\in (M,\infty )\cap k^{-1}\mathbb {N}}\left| \textrm{e}^{-\lambda x} H(x,m\lambda ) +B_k(x,\lambda )\right| \le \textrm{e}^{-(\lambda - \lambda _0)M} [K m\lambda + K_1\sup _{k \in \mathbb {N}} T_k(\lambda ) ]\sup _{x\ge 0} [(1+x)\textrm{e}^{-\lambda _0 x}], \end{aligned}$$

so (35) follows taking *M* large enough. $\square$

The proof of the convergence of the scaled processes is completed using essentially tightness arguments and the identification of a martingale problem. The next result is devoted to the former: establishing the tightness of the stochastic process sequence defined in (5).

### Theorem 9

Under Assumptions 1–7, the sequence of scaled processes $\{(z_k(t): t\in [0,\infty )):k\in \mathbb {N}\}$ is tight in $D([0,\infty ))$.

### Proof

The demonstration of the result is based on Aldous’ criterion, see [1, Theorem 1]. That is, to establish the tightness of the scaled processes, it is enough to verify the following two conditions. Firstly, for each fixed $t \ge 0$, the family $\{z_k(t): k \in \mathbb {N}\}$ is tight. Secondly, for every $T>0$, every sequence of stopping times $\tau _k \le T$, and every sequence of positive constants $\delta _k \rightarrow 0$ with $\tau _k + \delta _k \le T$, one has that $z_k(\tau _k+\delta _k) - z_k(\tau _k)$ converges in probability to zero as $k\rightarrow \infty$.

The proof is a convenient adaptation of the arguments in [17, Theorem 3.4]. We provide the corresponding scheme, focusing our attention in the steps requiring additional work.

First let us prove that

37 $$\begin{aligned} \sup _{k\in \mathbb {N}} \left( \mathbb {E} [z_k(\tau _k)] + \mathbb {E} [z_k(\tau _k+\delta _k)] \right) <\infty . \end{aligned}$$

Indeed, let us start by showing that

38 $$\begin{aligned} \sup _{k\in \mathbb {N}} \sup _{t\in [0,T]} \mathbb {E} [z_k(t)] < \infty . \end{aligned}$$

For $n\in \mathbb {N}_0$, we have

$$\begin{aligned} \mathbb {E}\left[ \frac{Z_k(n+1)}{k}\right]&= \mathbb {E}\left[ \frac{1}{k}\sum _{i=1}^{\phi _k^{(n)}(Z_k(n))} X_k^{n,i}\right] = \frac{\textrm{d}g_k}{\textrm{d}s}(1-) \mathbb {E}\left[ \frac{\phi _k^{(n)}(Z_k(n))}{k}\right] \\&= \frac{\textrm{d}g_k}{\textrm{d}s}(1-) \mathbb {E}\left[ \frac{\varphi _k^{(n)}(Z_k(n)) + \psi _k^{(n)}(Z_k(n))}{k}\right] . \end{aligned}$$

Recalling (7), we deduce that

$$\begin{aligned} \frac{\textrm{d}g_k}{\textrm{d}s}(1-) \le m \left( 1+\frac{K_2}{\gamma _k}\right) , \end{aligned}$$

and taking into account the *j*-divisibility of $\varphi _k^{(n)}(j)$ (Assumption 5), we get

$$\begin{aligned} \mathbb {E}\left[ \varphi _k^{(n)}(Z_k(n))\right] = \mathbb {E}\left[ Z_k(n) \frac{\textrm{d}f_k^{(Z_k(n))}}{\textrm{d}s}(1-)\right] , \end{aligned}$$

so

$$\begin{aligned} \frac{\textrm{d}g_k}{\textrm{d}s}(1-) \mathbb {E}\left[ \frac{\varphi _k^{(n)}(Z_k(n))}{k}\right]&\le \left( 1+\frac{K_2}{\gamma _k}\right) \mathbb {E}\left[ \frac{Z_k(n)}{k} m \frac{\textrm{d}f_k^{(Z_k(n))}}{\textrm{d}s}(1-)\right] \\&\le \left( 1+\frac{K_2}{\gamma _k}\right) \mathbb {E}\left[ \frac{Z_k(n)}{k} \left( 1 + \frac{1}{\gamma _k} \sup _{j\in \mathbb {N}} \left| \gamma _k \left( m \frac{\textrm{d}f_k^{(j)}}{\textrm{d}s}(1-)-1\right) \right| \right) \right] \\&\le \left( 1+\frac{K_2}{\gamma _k}\right) \left( 1+\frac{K_3}{\gamma _k}\right) \mathbb {E}\left[ \frac{Z_k(n)}{k}\right] , \end{aligned}$$

where $K_3$ was introduced in (10).

Likewise, we look for an upper bound for the other term of the control,

$$\begin{aligned} \mathbb {E}\left[ \psi _k^{(n)}(Z_k(n))\right]&= \sum _{j=0}^{\infty } \mathbb {E}\left[ \psi _k^{(n)} (j)\right] \mathbb {P}\left[ Z_k(n) = j\right] = \sum _{j=0}^{\infty } \frac{\textrm{d}h^{(j)}}{\textrm{d}s}(1-) \mathbb {P}\left[ Z_k(n) = j\right] \\&\le \sum _{j=0}^{\infty } \frac{k}{\gamma _k} K_1\left( 1+\frac{j}{k}\right) \mathbb {P}\left[ Z_k(n) = j\right] = \frac{k}{\gamma _k} K_1\left( 1+\mathbb {E}\left[ \frac{Z_k(n)}{k}\right] \right) , \end{aligned}$$

where we applied Assumption 4 in the inequality. Therefore,

$$\begin{aligned} \mathbb {E}\left[ \frac{Z_k(n+1)}{k}\right]&\le \left( 1+\frac{K_2}{\gamma _k}\right) \left( 1+\frac{K_3}{\gamma _k}\right) \mathbb {E}\left[ \frac{Z_k(n)}{k}\right] \\  &\quad + m \left( 1+\frac{K_2}{\gamma _k}\right) \frac{K_1}{\gamma _k} \mathbb {E}\left[ \frac{Z_k(n)}{k}\right] + m \left( 1+\frac{K_2}{\gamma _k}\right) \frac{K_1}{\gamma _k} \\&\le \left( 1+\frac{K_2}{\gamma _k}\right) \left( 1+\frac{K_3+mK_1}{\gamma _k}\right) \mathbb {E}\left[ \frac{Z_k(n)}{k}\right] + \left( 1+\frac{K_2}{\gamma _k}\right) \frac{mK_1}{\gamma _k} . \end{aligned}$$

Choosing $K_4> K_3 + mK_1$ and applying an induction argument, we get

$$\begin{aligned} \mathbb {E}\left[ \frac{Z_k(n+1)}{k}\right]&\le \left[ \left( 1+\frac{K_2}{\gamma _k}\right) \left( 1+\frac{K_4}{\gamma _k}\right) \right] ^{n+1} \mathbb {E}\left[ \frac{Z_k(0)}{k}\right] \\  &\quad + \sum _{j=0}^n \left( 1+\frac{K_2}{\gamma _k}\right) \frac{K_4}{\gamma _k} \left[ \left( 1+\frac{K_2}{\gamma _k}\right) \left( 1+\frac{K_4}{\gamma _k}\right) \right] ^{j} \\&= \left[ \left( 1+\frac{K_2}{\gamma _k}\right) \left( 1+\frac{K_4}{\gamma _k}\right) \right] ^{n+1} \mathbb {E}\left[ \frac{Z_k(0)}{k}\right] \\  &\quad + \left( 1+\frac{K_2}{\gamma _k}\right) \frac{K_4}{\gamma _k} \frac{\left[ \left( 1+\frac{K_2}{\gamma _k}\right) \left( 1+\frac{K_4}{\gamma _k}\right) \right] ^{n+1}-1}{\left( 1+\frac{K_2}{\gamma _k}\right) \left( 1+\frac{K_4}{\gamma _k}\right) - 1}. \end{aligned}$$

Then (38) holds because

$$\begin{aligned} \mathbb {E}\left[ z_k(t)\right]&\le \left[ \left( 1+\frac{K_2}{\gamma _k}\right) \left( 1+\frac{K_4}{\gamma _k}\right) \right] ^{\gamma _k T} \mathbb {E}\left[ \frac{Z_k(0)}{k}\right] \\  &\quad + \left( 1+\frac{K_2}{\gamma _k}\right) \frac{K_4}{\gamma _k} \frac{\left[ \left( 1+\frac{K_2}{\gamma _k}\right) \left( 1+\frac{K_4}{\gamma _k}\right) \right] ^{\gamma _k T}-1}{\left( 1+\frac{K_2}{\gamma _k}\right) \left( 1+\frac{K_4}{\gamma _k}\right) - 1} \end{aligned}$$

and

$$\begin{aligned} \lim _{k\rightarrow \infty }&\left[ \left( 1+\frac{K_2}{\gamma _k}\right) \left( 1+\frac{K_4}{\gamma _k}\right) \right] ^{\gamma _k T} \mathbb {E}\left[ \frac{Z_k(0)}{k}\right] + \left( 1+\frac{K_2}{\gamma _k}\right) \frac{K_4}{\gamma _k} \frac{\left[ \left( 1+\frac{K_2}{\gamma _k}\right) \left( 1+\frac{K_4}{\gamma _k}\right) \right] ^{\gamma _k T}-1}{\left( 1+\frac{K_2}{\gamma _k}\right) \left( 1+\frac{K_4}{\gamma _k}\right) - 1} \\&\quad = \textrm{e}^{(K_2+K_4)T} \cdot 0 + 1 \cdot K_4T = K_4T. \end{aligned}$$

Let $\textrm{id}$ be the identity function. Recalling the generator $A_k$, we see that

$$\begin{aligned} A_k\textrm{id}(x)&= \gamma _k \mathbb {E} \left[ \textrm{id}(k^{-1} Z_k(j+1)) \mid k^{-1} Z_k(j)=x\right] - \gamma _k \textrm{id}(x) \\&= \gamma _k\left( \mathbb {E}\left[ \frac{1}{k}\sum _{i=1}^{\phi _k^{(n) }(kx)}X_k^{n,i} \right] -x\right) . \end{aligned}$$

Hence

$$\begin{aligned} |A_k \textrm{id}(x) |&= \gamma _k\left| \frac{\textrm{d}g_k}{\textrm{d}s}(1-)\mathbb {E}\left[ \frac{\phi _k^{(n) }(kx)}{k}\right] -x\right| = \gamma _k\left| \frac{\textrm{d}g_k}{\textrm{d}s}(1-)\frac{\mathbb {E}\left[ \varphi _k^{(n) }(kx)\right] +\mathbb {E}\left[ \psi _k^{(n) }(kx)\right] }{k} -x\right| \\&= \gamma _k\left| \frac{\textrm{d}g_k}{\textrm{d}s}(1-)\frac{\frac{\textrm{d}h_k^{(kx)}}{\textrm{d}s}(1-)+kx \frac{\textrm{d}f_k^{(kx)}}{\textrm{d}s}(1-)}{k} -x\right| . \end{aligned}$$

By Remark 2 and Assumptions 4 and 6, there exists a positive constant $K_5$ such that

$$\begin{aligned} |A_k \textrm{id}(x) |&\le \frac{\textrm{d}g_k}{\textrm{d}s}(1-) K_1 (1+x)+ x \gamma _k \left| \frac{\textrm{d}g_k}{\textrm{d}s}(1-)\frac{\textrm{d}f_k^{(kx)}}{\textrm{d}s}(1-)-1\right| \\&\le \frac{\textrm{d}g_k}{\textrm{d}s}(1-) K_1 (1+x)+ x \gamma _k \left| \frac{1}{m}\frac{\textrm{d}g_k}{\textrm{d}s}(1-)-1\right| + x \frac{1}{m}\frac{\textrm{d}g_k}{\textrm{d}s}(1-)\gamma _k \left| m\frac{\textrm{d}f_k^{(kx)}}{\textrm{d}s}(1-)-1\right| \\&\le K_5 (1+x). \end{aligned}$$

For a bounded and measurable real function *f* on $[0,\infty )$, we now define

39 $$\begin{aligned} M_k^{f} (n) = \gamma _k f(k^{-1} Z_k(n)) - \gamma _k f (k^{-1} Z_k(0)) - \sum _{i=0}^{n-1} A_kf(k^{-1} Z_k(i)). \end{aligned}$$

It is easy to check that $M_k^{f} (n)$ is a martingale with respect to $\sigma (Z_k(0),\ldots ,Z_k(n))$. Consequently,

$$\begin{aligned} 0 = \mathbb {E}\left[ M_k^{\textrm{id}} (0)\right] = \mathbb {E}\left[ M_k^{\textrm{id}} (\lfloor \gamma _k t\rfloor )\right] = \gamma _k \mathbb {E}\left[ z_k(t)\right] - \gamma _k \mathbb {E}\left[ z_k(0)\right] - \mathbb {E}\left[ \sum _{i=0}^{\lfloor \gamma _k t\rfloor -1} A_k\textrm{id}(k^{-1} Z_k(i))\right] , \end{aligned}$$

or equivalently,

$$\begin{aligned} \mathbb {E}\left[ z_k(t)\right] = \mathbb {E}\left[ z_k(0)\right] + \mathbb {E}\left[ \sum _{i=0}^{\lfloor \gamma _k t\rfloor -1} \gamma _k^{-1} A_k\textrm{id}(k^{-1} Z_k(i))\right] = \mathbb {E}\left[ z_k(0)\right] + \mathbb {E}\left[ \int _0^{\lfloor \gamma _k t\rfloor /\gamma _k} A_k\textrm{id}(z_k(s)) \textrm{d}s\right] . \end{aligned}$$

Finally, by Doob’s Stopping Theorem, we get

$$\begin{aligned} \mathbb {E}\left[ z_k(\tau _k)\right]&\le \mathbb {E}\left[ z_k(0)\right] + \mathbb {E}\left[ \int _0^{\tau _k}A_k \textrm{id}(z_k(t))\textrm{d}t\right] \\&\le \mathbb {E}\left[ z_k(0)\right] + \int _0^{T}K_5 \mathbb {E}\left[ 1+z_k(t)\right] \textrm{d}t \\&\le \sup _{k\in \mathbb {N}} \mathbb {E}\left[ z_k(0)\right] + TK_5 (1+\sup _{k\in \mathbb {N}}\sup _{t\in [0,T]}\mathbb {E}\left[ z_k(t)\right] ). \end{aligned}$$

From (38) and Assumption 7, we see that $\sup _{k\in \mathbb {N}} \mathbb {E}\left[ z_k(\tau _k)\right] <\infty .$ The same argument leads to $\sup _{k\in \mathbb {N}} \mathbb {E}\left[ z_k(\tau _k+\delta _k)\right] <\infty$ and then (37) follows.

As a consequence of (38), we also have that $\{z_k(t): k\in \mathbb {N}\}$ is tight for a fix $t\ge 0$.

Finally, it can be proved as in [17, Lemma 3.2 and Theorem 3.4], making use of Theorem 8, that for $\lambda >0$,

$$\begin{aligned} \lim _{k\rightarrow \infty }\mathbb {E}\left[ |e_\lambda (z_k(\tau _k+\delta _k))-e_\lambda (z_k(\tau _k))|^2\right] =0, \end{aligned}$$

and hence, for any constant $M>0$ and $\epsilon >0$,

$$\begin{aligned} \lim _{k\rightarrow \infty }\mathbb {P}(|z_k(\tau _k+\delta _k)-z_k(\tau _k)|>\epsilon , \max \{z_k(\tau _k+\delta _k),z_k(\tau _k)\}\le M)=0. \end{aligned}$$

Considering (37) and the fact that

$$\begin{aligned} \mathbb {P}(|z_k(\tau _k+\delta _k)-z_k(\tau _k)|>\epsilon )&\le \mathbb {P}(|z_k(\tau _k+\delta _k)-z_k(\tau _k)|>\epsilon , \max \{z_k(\tau _k+\delta _k),z_k(\tau _k)\}\le M)\\&\quad +\mathbb {P}(z_k(\tau _k+\delta _k)\ge M)+\mathbb {P}(z_k(\tau _k)\ge M), \end{aligned}$$

we deduce the convergence in probability of $z_k(\tau _k+\delta _k)-z_k(\tau _k)$ as $k,M\rightarrow \infty$. Therefore, we showed the tightness of the scaled processes. $\square$

The work above now puts us in a position to conclude with the proof of the main result of the paper.

### Proof of Theorem 1

Taking the previous results into account, the main theorem is proved using reasoning similar to that used in [17, Theorem 4.2]. By Theorem 9, $\{(z_k(t): t\in [0,\infty )):k\in \mathbb {N}\}$ is relatively compact. Hence, it is possible to find a subsequence $\{(z_{k_i}(t): t\in [0,\infty )):k_i\in \mathbb {N}\}$ converging to a càdlàg process. That is, there exists a probability measure *Q* on $D([0,\infty ))$ such that $Q=\lim _{i\rightarrow \infty }P^{(k_i)}$, with $P^{(k_i)}$ being the distribution of process $(z_{k_i}(t): t\in [0,\infty ))$ in $D([0,\infty ))$. By Skorokhod’s representation theorem, see for instance [2, Theorem 6.7], there exists a probability space $(\Omega ', \mathcal {F}', P')$ supporting càdlàg processes $(x(t): t \ge 0)$ and $(x_{k_i}(t): t \ge 0)$ whose laws on $D([0,\infty ))$ are *Q* and $P^{(k_i)}$, respectively, and such that $x_{k_i} \rightarrow x$ $P'$-a.s. From this, using [4, Proposition 5.2, Chapter 3], we obtain that $x_{k_i}(t)$ converges to *x*(*t*) for all continuity points *t* of $(x(t):t\in [0,\infty ))$. We recall that the set of discontinuity points of this process is at most countable, see [4, Lemma 7.7, Chapter 3].

Now, we use the relationship between weak solutions of equation (11) and the associated martingale problem (see [17, Theorem 4.1]). Specifically, a positive càdlàg process $(z(t): t\in [0,\infty ))$ is a weak solution of (11) with initial value *z*(0) if and only if

40 $$\begin{aligned} f\!\left( z(t)\right) = f\!\left( z(0)\right) + \int _0^t A f\!\left( z(s)\right) \textrm{d}s + \text {local martingale}, \qquad t \ge 0, \quad f \in C_c^2([0,\infty )). \end{aligned}$$

Let *P* denote the distribution of $(z(t):t\in [0,\infty ))$ in $D([0,\infty ))$. If we prove that $(x(t): t\in [0,\infty ))$ solves the martingale problem (40), then it is a weak solution of (11), and the pathwise uniqueness of (11) implies that $Q=P$, and therefore the proof of Theorem 1 is completed.

Indeed, let us consider (39) with $f=e_{\lambda }$, which we can rewrite as

41 $$\begin{aligned} e_\lambda (x_{k_i}(t)) = e_\lambda (x_{k_i}(0)) + \int _0^{\lfloor \gamma _{k_i} t \rfloor / \gamma _{k_i}} A_{k_i} e_\lambda (x_{k_i}(s))\, \textrm{d}s + \gamma _{k_i}^{-1} M^{e_\lambda }_{k_i}(\lfloor \gamma _{k_i} t \rfloor ). \end{aligned}$$

Taking into account that

$$\begin{aligned} \int _0^{\lfloor \gamma _{k_i} t \rfloor / \gamma _{k_i}} \left| A_{k_i} e_\lambda (x_{k_i}(s)) - A e_\lambda (x(s)) \right| \,\textrm{d}s&\le \int _0^{\lfloor \gamma _{k_i} t \rfloor / \gamma _{k_i}} \left| A_{k_i} e_\lambda (x_{k_i}(s)) - A e_\lambda (x_{k_i}(s)) \right| \,\textrm{d}s \\&\quad + \int _0^{\lfloor \gamma _{k_i} t \rfloor / \gamma _{k_i}} \left| A e_\lambda (x_{k_i}(s)) - A e_\lambda (x(s)) \right| \,\textrm{d}s, \end{aligned}$$

and using Theorem 8 together with the considerations made in the first paragraph of this proof, we can take the limit as $i \rightarrow \infty$ in (41). By the dominated convergence theorem, it follows that

42 $$\begin{aligned} M^f(t) = f(x(t)) - f(x(0)) - \int _0^t A f(x(s))\,\textrm{d}s, \qquad \text {with } f = e_\lambda , \end{aligned}$$

is a martingale. For $f \in C_c^2([0,\infty ))$, and considering Remark 5, let $\{f_n\}$ be a sequence of functions in the linear span of $\{e_{\lambda }: \lambda \ge 0\}$ such that $\lim _{n\rightarrow \infty }f_n=f$, $\lim _{n\rightarrow \infty }f'_n=f'$ and $\lim _{n\rightarrow \infty }f''_n=f''$ uniformly on $[0,\infty )$, so that $\lim _{n\rightarrow \infty } A f_n(x) = A f(x)$ uniformly on each bounded interval. From (42),

43 $$\begin{aligned} f_n(x(t)) = f_n(x(0)) + \int _0^t A f_n(x(s))\textrm{d}s + M^{f_n}(t), \end{aligned}$$

where $M^{f_n}$ is a martingale. Let $\widetilde{\tau }_M := \inf \{ t > 0 : x(t) \ge M \}$. Since $(x(t):t\in [0,\infty ))$ is a càdlàg process, $\lim _{M\rightarrow \infty }\widetilde{\tau }_M = \infty$ a.s. Considering $t \wedge \widetilde{\tau }_M$ instead of *t*, and taking limit in (43), we have that

$$ M^f (t \wedge \widetilde{\tau }_M) = f(x(t\wedge \widetilde{\tau }_M)) - f(x(0)) - \int _0^t A f(x(s \wedge \widetilde{\tau }_{M^-}))\textrm{d}s $$

is a martingale. Therefore, we conclude that $(x(t): t\in [0,\infty ))$ solves the martingale problem (40). $\square$

## Data Availability

Data sharing is not applicable to this article as no datasets were generated or analysed during the current study.

## Appendix A Proof of Lemmas 3 and 4

We must first introduce the concept of complete monotonicity for a function.

### Definition 2

A function $\theta \in C([0,\infty ))$ is called completely monotone if

$$\begin{aligned} (-1)^j \Delta _d^j \theta (\lambda ) \ge 0, \qquad \lambda \in [0,\infty ), \end{aligned}$$

for every $j\in \mathbb {N}_0$ and $d\in [0,\infty )$, where $\Delta _d^0$ is the identity operator, $\Delta _d^j = \Delta _d^{j-1} \Delta _d$ is defined recursively for $j\in \mathbb {N}$ and $\Delta _d$ is the difference operator

$$\begin{aligned} \Delta _d f (\lambda ) = f (\lambda +d) - f (\lambda ), \qquad \lambda +d, \lambda \in I, \end{aligned}$$

where *I* is an interval in $\mathbb {R}$ and *f* is a continuous function in *I*.

We point out below that the successive difference operators introduced in the previous definition are closely related to the order-corresponding derivatives.

### Remark 6

If $$f\in C^\infty (I)$$, then

$$\begin{aligned} \frac{\textrm{d}^n f}{\textrm{d}\lambda ^n}(\lambda ) = \lim _{h\rightarrow 0+} \frac{\Delta _h^n f(\lambda )}{h^n}, \qquad \lambda \in I, \quad n\in \mathbb {N}_0, \end{aligned}$$

and, by mean-value theorem, for all $\lambda , h \in [0,\infty )$ there exists $\tilde{\lambda }\in [\lambda , \lambda +h]$ such that

$$\begin{aligned} \Delta _h^n f(\lambda ) = h^n \frac{\textrm{d}^n f}{\textrm{d}\lambda ^n}(\tilde{\lambda }). \end{aligned}$$

We present now the proof of Lemma 3.

### Proof of Lemma 3

Firstly, let us show that the function *G* has representation

A1 $$\begin{aligned} G(\lambda ) = a_0 + a_1 \lambda + \int _0^\infty \left( \textrm{e}^{-\lambda u} - 1 + \frac{\lambda u}{1+u^2}\right) \left( 1-\textrm{e}^{-u}\right) ^{-2} L(\textrm{d}u), \end{aligned}$$

for all $\lambda \in [0,\infty )$, where $a_0,a_1\in \mathbb {R}$, $L(\textrm{d}u)$ is a finite measure on $[0,\infty )$ and the integrand in (A1) is defined at $u=0$ by continuity as

$$\begin{aligned} \lim _{u\rightarrow 0} \left( \textrm{e}^{-\lambda u} - 1 + \frac{\lambda u}{1+u^2}\right) \left( 1-\textrm{e}^{-u}\right) ^{-2}&= \lim _{u\rightarrow 0} \left( \frac{\lambda ^2u^2}{2} -\lambda u + \frac{\lambda u}{1+u^2}\right) u^{-2} \\&= \lim _{u\rightarrow 0} \left( \frac{\lambda ^2}{2} - \frac{\lambda u}{1+u^2}\right) = \frac{\lambda ^2}{2}. \end{aligned}$$

By the main theorem from [12], the function *G*, which is continuous as noted in Remark 1, has representation (A1) if and only if for every $c\ge 0$ the function $\Delta _c^2 G$ is completely monotone. For any $j\in \mathbb {N}_0$, $c\in [0,\infty )$, $d\in [0,\infty )$, $\lambda \in [0,\infty )$ and sufficiently large $k\in \mathbb {N}$, $(-1)^j \Delta _d^j \Delta _c^2 G_k(\lambda )$ is well defined and

$$\begin{aligned} (-1)^j \Delta _d^j \Delta _c^2 G(\lambda ) = \lim _{k\rightarrow \infty } (-1)^j \Delta _d^j \Delta _c^2 G_k(\lambda ). \end{aligned}$$

Thus $\Delta _c^2 G$ is completely monotone if we prove that $(-1)^j \Delta _d^j \Delta _c^2 G_k(\lambda ) \ge 0$. From Remark 6, it is enough to see that

$$\begin{aligned} (-1)^j \frac{\textrm{d}^j \Delta _c^2 G_k}{\textrm{d}\lambda ^j}(\lambda ) \ge 0. \end{aligned}$$

Indeed, we have

$$\begin{aligned} \Delta _c G_k(\lambda ) = \frac{k\gamma _k}{m}\left[ \Delta _c g_k\bigg (1-\frac{\cdot }{k}\bigg )(\lambda )+\frac{mc}{k}\right] , \end{aligned}$$

$$\begin{aligned} \Delta _c^2 G_k(\lambda ) = \frac{k\gamma _k}{m}\left[ \Delta _c^2 g_k\bigg (1-\frac{\cdot }{k}\bigg )(\lambda )\right] , \end{aligned}$$

$$\begin{aligned} (-1)^j \frac{\textrm{d}^j \Delta _c^2 G_k}{\textrm{d}\lambda ^j}(\lambda ) = \frac{k\gamma _k}{m} \frac{1}{k^j} \Delta _c^2 \frac{\textrm{d}^j g_k}{\textrm{d}\lambda ^j}\bigg (1-\frac{\cdot }{k}\bigg )(\lambda ). \end{aligned}$$

Now, we apply mean-value theorem to

$$\begin{aligned} \Delta _c^2 \frac{\textrm{d}^j g_k}{\textrm{d}\lambda ^j}\bigg (1-\frac{\cdot }{k}\bigg )(\lambda )&= \left[ \frac{\textrm{d}^j g_k}{\textrm{d}\lambda ^j}\bigg (1-\frac{\lambda }{k}\bigg ) - \frac{\textrm{d}^j g_k}{\textrm{d}\lambda ^j}\bigg (1-\frac{\lambda +c}{k}\bigg )\right] \\&\quad - \left[ \frac{\textrm{d}^j g_k}{\textrm{d}\lambda ^j}\bigg (1-\frac{\lambda +c}{k}\bigg ) - \frac{\textrm{d}^j g_k}{\textrm{d}\lambda ^j}\bigg (1-\frac{\lambda +2c}{k}\bigg )\right] , \end{aligned}$$

then there exist $s_1\in [1-(\lambda +c)/k,1-\lambda /k]$ and $s_2\in [1-(\lambda +2c)/k,1-(\lambda +c)/k]$ such that

$$\begin{aligned} \Delta _c^2 \frac{\textrm{d}^j g_k}{\textrm{d}\lambda ^j}\bigg (1-\frac{\cdot }{k}\bigg )(\lambda ) = \frac{c}{k} \frac{\textrm{d}^{j+1} g_k}{\textrm{d}\lambda ^{j+1}}(s_1) -\frac{c}{k} \frac{\textrm{d}^{j+1} g_k}{\textrm{d}\lambda ^{j+1}}(s_2). \end{aligned}$$

But $\textrm{d}^j g_k / \textrm{d}\lambda ^j$ is a power series with non-negative coefficients (in particular it is monotically increasing for all *j*) because $g_k$ is a PGF, so

$$\begin{aligned} \Delta _c^2 \frac{\textrm{d}^j g_k}{\textrm{d}\lambda ^j}\bigg (1-\frac{\cdot }{k}\bigg )(\lambda ) \ge \frac{c}{k} \left[ \frac{\textrm{d}^{j+1} g_k}{\textrm{d}\lambda ^{j+1}}\bigg (1-\frac{\lambda +c}{k}\bigg ) - \frac{\textrm{d}^{j+1} g_k}{\textrm{d}\lambda ^{j+1}}\bigg (1-\frac{\lambda +c}{k}\bigg )\right] = 0. \end{aligned}$$

As a result, representation (A1) is proven, in fact, $a_0=0$ due to $G(0)=a_0$ and $G_k(0) = 0$. This representation can be equivalently reformulated as

A2 $$\begin{aligned} G(\lambda ) = b_1 \lambda + b_2 \lambda ^2 + \int _0^\infty \left( \textrm{e}^{-\lambda u} - 1 + \frac{\lambda u}{1+u^2}\right) \mu (\textrm{d}u), \qquad \lambda \in [0,\infty ), \end{aligned}$$

where $b_1\in \mathbb {R}$, $b_2\in [0,\infty )$ and $\mu (\textrm{d}u)$ is a $\sigma$-finite measure on $(0,\infty )$ with

$$\begin{aligned} \int _0^\infty \left( 1\wedge u^2\right) \mu (\textrm{d}u) <\infty . \end{aligned}$$

The equivalence in the rewriting follows from $a_1 = b_1$ and $L(\textrm{d}u ) = (1-\textrm{e}^{-u})^2 \mu (\textrm{d}u) + 2b_2 \delta _0 (\textrm{d}u)$, where $\delta _0$ is the Dirac measure for 0.

Finally, as we stated in Remark 1, *G* is locally Lipschitz, then by [16, Proposition 1.48] the measure $\mu (\textrm{d}u)$ satisfies

$$\begin{aligned} \int _0^\infty \left( u\wedge u^2\right) \mu (\textrm{d}u) <\infty , \end{aligned}$$

and representation (A2) can be written as a branching mechanism (3) with

$$\begin{aligned} a = b_1 - \int _0^\infty \frac{u^3}{1+u^2} \mu (\textrm{d}u) \end{aligned}$$

and $$b = \sqrt{2b_2}.$$ $$\square $$

To conclude, we prove Lemma 4.

### Proof of Lemma 4

We use a parallel logic to the steps of Proposition 2.6 in [15]. We will decompose the target limit function $G(\lambda )$ into two parts, separating the linear term from the quadratic and integral terms. Let us start with the later denoting

$$\begin{aligned} G_0(\lambda ) = G(\lambda ) - a\lambda - \frac{1}{2} b^2 \lambda ^2 = \int _0^\infty \left( \textrm{e}^{-\lambda u}-1+\lambda u\right) \mu (\textrm{d}u). \end{aligned}$$

The idea is to construct a sequence of functions $\{G_{0,k}\}_{k\in \mathbb {N}}$ converging to the the quadratic and integral terms of *G*. We do this by using the PGF of a Poisson distribution with parameter *m*. For all $m>0$, choosing

$$\begin{aligned} \gamma _{0,k} = \frac{b^2}{m}k + \textrm{e}^m \int _0^\infty u\left( 1-\textrm{e}^{-ku}\right) \mu (\textrm{d}u) \end{aligned}$$

and

$$\begin{aligned} g_{0,k}(s) = \textrm{e}^{m(s-1)} + \frac{m}{k\gamma _{0,k}}G_0(k(1-s)), \qquad |s| \le 1, \end{aligned}$$

it is easy to see that the sequence of functions

$$\begin{aligned} G_{0,k}(\lambda ) = \frac{k\gamma _{0,k}}{m}\left[ g_{0,k}\bigg (1-\frac{\lambda }{k}\bigg )-\left( 1-\frac{m\lambda }{k}\right) \right] = \frac{k\gamma _{0,k}}{m}\left[ \textrm{e}^{-m\lambda /k} - \left( 1-\frac{m\lambda }{k}\right) \right] + G_0(\lambda ) \end{aligned}$$

is uniformly Lipschitz on bounded intervals and converges uniformly to $\frac{1}{2}b^2\lambda ^2 + G_0(\lambda )$ on bounded intervals. Let us simply check that $g_{0,k}$ is a valid PGF. Indeed, $g_{0,k}(1) = 1$, $g_{0,k}(0) = \textrm{e}^{-m} + \frac{m}{k\gamma _{0,k}}G_0(k) \ge 0$ and their derivatives at $s=0$ are non-negative due to our choice of $\gamma _{0,k}$:

$$\begin{aligned} \frac{\textrm{d}g_{0,k}}{\textrm{d}s} (0) = m\textrm{e}^{-m} - \frac{m}{k\gamma _{0,k}} \int _0^\infty ku\left( 1- \textrm{e}^{-ku}\right) \mu (\textrm{d}u) \ge 0, \end{aligned}$$

$$\begin{aligned} \frac{\textrm{d}^n g_{0,k}}{\textrm{d}s^n}(0) = m^n\textrm{e}^{-m} + \frac{m}{k\gamma _{0,k}} k^n \int _0^\infty u^n \textrm{e}^{-ku}\mu (\textrm{d}u) \ge 0, \qquad n\ge 2. \end{aligned}$$

Now we address the linear term. If $a = 0$, the proof is complete. If $a \ne 0$, we construct a sequence $G_{1,k}(\lambda )$ converging to $a\lambda$. We need an integer power $n > m$ to ensure we can handle negative drift, let us say $n = \lfloor m \rfloor + 2$. Defining

$$\begin{aligned} \gamma _{1,k}&= {\left\{ \begin{array}{ll} a &  \text {if } a> 0, \\ \frac{m|a|}{n-m} &  \text {if } a< 0, \end{array}\right. } \\ g_{1,k}(s)&= {\left\{ \begin{array}{ll} 1 &  \text {if } a > 0, \\ s^n &  \text {if } a < 0, \end{array}\right. }\quad \text {and}\quad G_{1,k}(\lambda ) = \frac{k\gamma _{1,k}}{m}\left[ g_{1,k}\bigg (1-\frac{\lambda }{k}\bigg )-\left( 1-\frac{m\lambda }{k}\right) \right] ,\end{aligned}$$

it is easy to check that $G_{1,k}(\lambda )$ converges to $a\lambda$.

Finally, we combine the two parts defining

$$\begin{aligned} \gamma _k = \gamma _{0,k} + \gamma _{1,k} \qquad \text {and} \qquad g_k = \frac{\gamma _{0,k}g_{0,k} + \gamma _{1,k}g_{1,k}}{\gamma _k}. \end{aligned}$$

By construction, $g_k$ is a valid PGF. Let us define $G_k$ by (6) with $\gamma _k$ and $g_k$ given above. Then $G_k=G_{0,k} + G_{1,k}$ satisfies Assumption 2. $\square$
